# Ultrasound modulates ion channel currents

**DOI:** 10.1038/srep24170

**Published:** 2016-04-26

**Authors:** Jan Kubanek, Jingyi Shi, Jon Marsh, Di Chen, Cheri Deng, Jianmin Cui

**Affiliations:** 1 Department of Molecular and Cellular Physiology, 279 Campus Drive, Stanford University, Stanford, CA 94305, USA; 2 Department of Biomedical Engineering, One Brookings Dr., Washington University in St. Louis, St. Louis, MO 63130, USA; 3 Department of Biomedical Engineering, 2200 Bonisteel Blvd.,University of Michigan, Ann Arbor, MI 48109, USA; 4 Department of Internal Medicine, 4320 Forest Park Ave, Washington University, St. Louis, MO 63110, USA

## Abstract

Transcranial focused ultrasound (US) has been demonstrated to stimulate neurons in animals and humans, but the mechanism of this effect is unknown. It has been hypothesized that US, a mechanical stimulus, may mediate cellular discharge by activating mechanosensitive ion channels embedded within cellular membranes. To test this hypothesis, we expressed potassium and sodium mechanosensitive ion channels (channels of the two-pore-domain potassium family (K2P) including TREK-1, TREK-2, TRAAK; Na_V_1.5) in the *Xenopus* oocyte system. Focused US (10 MHz, 0.3–4.9 W/cm^2^) modulated the currents flowing through the ion channels on average by up to 23%, depending on channel and stimulus intensity. The effects were reversible upon repeated stimulation and were abolished when a channel blocker (ranolazine to block Na_V_1.5, BaCl_2_ to block K2P channels) was applied to the solution. These data reveal at the single cell level that focused US modulates the activity of specific ion channels to mediate transmembrane currents. These findings open doors to investigations of the effects of  US on ion channels expressed in neurons, retinal cells, or cardiac cells, which may lead to important medical applications. The findings may also pave the way to the development of sonogenetics: a non-invasive, US-based analogue of optogenetics.

Focused ultrasound (US) has recently sparked attention in the therapeutic and interventional domains. Of particular interest, it has been demonstrated that US is capable of modulating neuronal activity^1^,^2^,^3^. The application of US elicits discharge activity in neurons and retinal cells with short latency^4^,^5^,^6^, triggers limb or eye movements upon the stimulation of motor circuits in animals^5^,^7^,^8^,^9^,^10^, and appears to elicit tactile sensations upon the stimulation of somatosensory cortex in humans^11^.

A major advantage of using US to control cellular discharge is that US readily propagates through biological tissues including the skull^2^,^3^,^11^,^12^ and can therefore be applied non-invasively. Further, US can be focused into a region of interest at depth without affecting intervening tissue layers. For stimulation of brain circuits, the ultrasonic

approach thus offers a sharper spatial focus and a deeper penetration compared to alternative non-invasive approaches such as the transcranial magnetic stimulation (TMS) or transcranial electrical stimulation (tACS, tDCS)^13^.

These favorable properties may position US as a new non-invasive tool to investigate the contribution of particular brain circuits to particular behaviors. Further, the approach may lead to important clinical applications. For instance, US could complement or substitute TMS in the treatment of depression, anxiety, or migraines^14^. US could also be used as a non-invasive alternative to deep brain stimulation to alleviate motor symptoms of advanced Parkinson’s disease. Such non-invasive approaches would bypass the associated surgeries^15^,^16^. Moreover, US could be applied to treat status epilepticus, a direction that has shown promise in rodents^2^,^17^. Researchers have also considered US for diagnosis of diseased retinal cells and as a driving element in future retinal prostheses^6^. In the cardiac tissue, US may have defibrillatory and antiarrhythmic effects^18^,^19^. Pace-making effects have also been demonstrated^19^,^20^.

To bring the approach to such applications, it is crucial to first infer the mechanism of the US action on excitable cells. Which types of cells modulate their activity by US? And what stimulus parameters should be used? To date, the mechanisms of US action on cellular excitability is poorly understood. There are several possibilities being considered.

First, the effects may be due to heating. However, only minimal changes in temperature have been computed and measured during US applications for neuromodulation^5^,^6^,^7^. Second, US may lead to cavitation, a phenomenon characterized by formation and collapse of gaseous bodies in liquid media or soft tissues. The likelihood of inception of cavitation increases with lower frequencies (sub-MHz or lower) and with higher acoustic pressures^8^,^21^. However, neuromodulatory effects have been observed even at the frequency of 43 MHz, at which the cavitation threshold is significantly higher than the levels used^6^. Third, it was proposed that US may separate the leaflets of lipid membranes^22^, which may lower membrane capacitance^23^. However, no appreciable changes in membrane capacitance have been detected^24^,^25^. Fourth, it has been hypothesized that US may affect the activity of mechanosensitive ion channels^4^,^26^. In particular, US may stretch or displace cellular membrane to alter the state of mechanosensitive ion channels embedded within and so mediate transmembrane currents. This hypothesis has not been directly tested.

To test this hypothesis, we measured the electric currents flowing through mechano-sensitive ion channels expressed in a single cell in response to US. We probed the activity of two-pore-domain potassium family (K2P) members including TREK-1 (hK2P2.1, KCNK2), TREK-2 (hK2P10.1, KCNK10), and TRAAK (hK2P4.1, KCNK4), which are expressed in neurons and retinal cells^27^,^28^,^29^,^30^,^31^ and are known to respond to positive and negative pressures applied to the membrane^27^,^32^,^33^. We also investigated the US response of Na_V_1.5 (SCN5A), a Na^+^-selective voltage-activated ion channel expressed in cardiomyocytes, neurons, and other excitable cells^34^,^35^,^36^,^37^, and also showing mechanosensitive responses^36^,^38^,^39^,^40^. We used acoustic parameters comparable to those applied to activate neurons or retinal cells in previous studies^6^,^8^.

## Results

**Figure 1:**
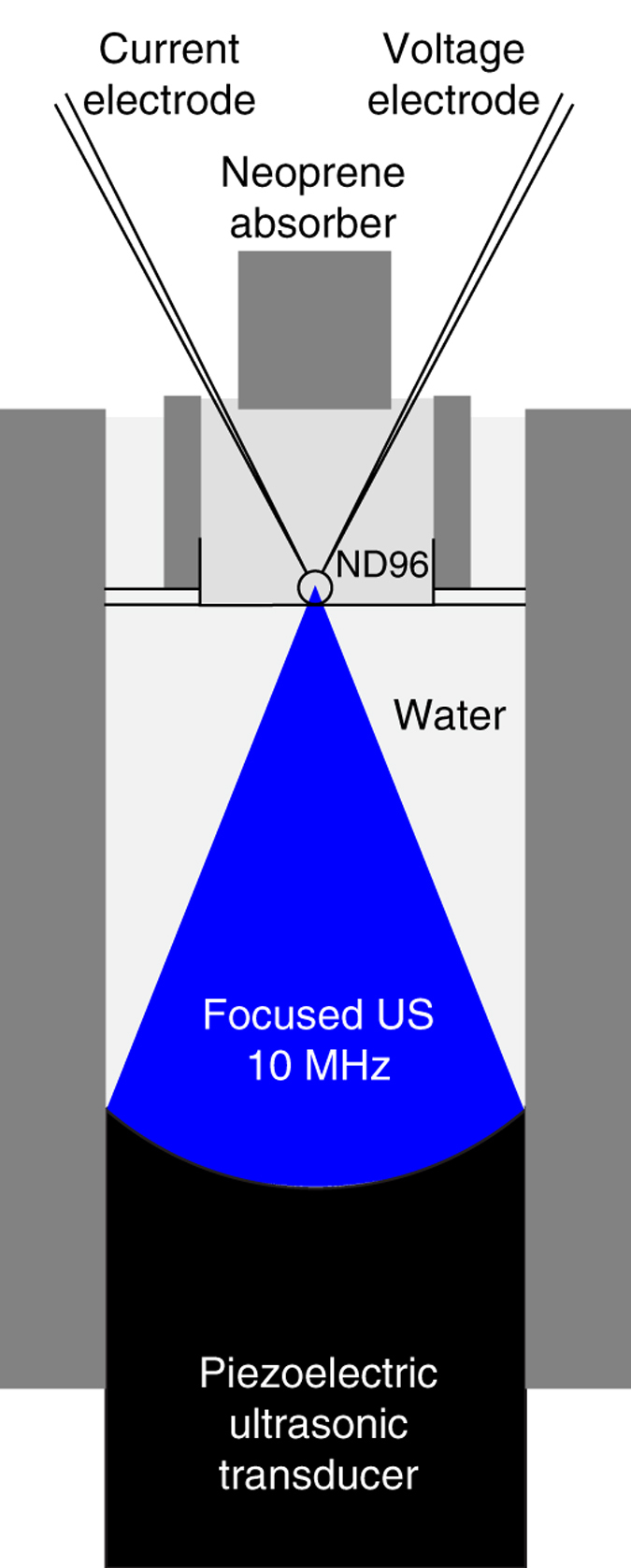
Measurement of currents flowing through ion channels in response to ultrasonic stimulation. A *Xenopus* oocyte expressing an ion channel of interest is placed in a recording bath in a chamber (see Methods), and subjected to an US pressure field generated by a focused US transducer placed below the chamber and submerged in deionized and degassed water. A neoprene absorber is immersed in the recording bath to prevent the formation of a standing wave in the chamber. Transmembrane currents are measured using the two-electrode voltage clamp.

We expressed mechanosensitive ion channels in *Xenopus* oocytes (see Methods for details), and subjected each single cell to a focused US stimulus (Fig. 1). The currents flowing through the cell membrane were measured using the two-electrode voltage clamp (Methods).

**Figure 2:**
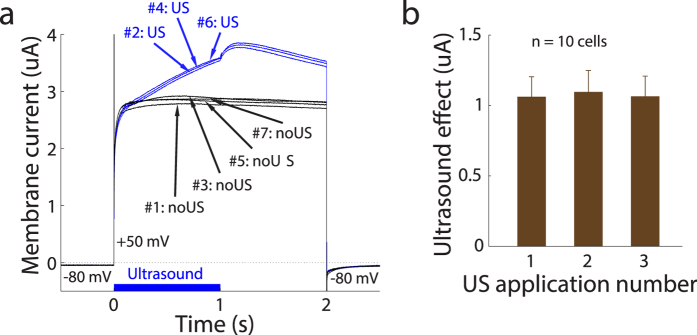
Robust and repeatable effects of ultrasound on transmembrane currents flowing through cells expressing TREK-1. (**a**) The current flowing through the membrane of an example cell as a function of time. The membrane voltage was held at −80 mV before stepping to +50 mV (time 0). After 2 s, the membrane voltage returned to −80 mV. To test the repeatability of the US effects, we alternated conditions without and with US in the following sequence: #1: noUS, #2: US, #3: noUS, #4: US, #5: noUS, #6: US, #7: noUS with a 5 s period between the repetitions. The corresponding current waveforms are labeled in the figure. In the US conditions, the US was applied at time 0 and lasted 1 s (see blue bar on the bottom). The driving US waveform was sinusoidal with frequency of 10 MHz and spatial-peak pressure amplitude of 240 kPa (2.0 W/cm^2^). The figure shows the raw recorded currents. (**b**) Mean ± s.e.m. ultrasound effect, i.e., US minus noUS currents, for the three consecutive applications of the US. The currents were measured between 1.0 s to 1.2 s following the stimulus onset. This plot is based on 10 cells using spatial-peak US pressure amplitude of 240 kPa (2.0 W/cm^2^) and membrane voltage of +50 mV.

We first tested the responses of TREK-1 of the K2P family. Figure 2a shows example currents flowing through the membrane of a representative cell as a function of time. Before time 0, the cell was held at −80 mV. At time 0, the membrane voltage stepped to +50 mV, for 2 s before returning back to −80 mV. When no US was applied (#1: noUS), the voltage step elicited a substantial current (about 2.8 uA) flowing out of the cell. This effect is as expected due to voltage-dependent changes of TREK-1 activation^33^,^41^. The effect quickly vanished when returning to −80 mV, in line with previous reports^33^,^41^.

Critically, in the next iteration, we investigated the effect of US (10 MHz, 240 kPa amplitude (2.0 W/cm^2^, see Methods)) on the transmembrane current. The US was applied starting at time 0 and lasted for 1 s (see blue bar on the bottom of Fig. 2a). The figure demonstrates that the US stimulus (#2: US) had a significant impact on the transmembrane current. The effect gradually increased with time. Following the offset of the US (time = 1 s), there was an additional current increase transient. The US effect then progressively decreased in amplitude, and vanished suddenly upon returning to the −80 mV membrane voltage. During the next iteration with no US applied (#3: noUS), the current was back at its pre-US level. These effects were robust and repeatable, as demonstrated by the individual traces in Fig. 2a.

We quantified the repeatability of the US effects (Fig. 2b). In this and following figures, currents were measured in the period from 1.0 s to 1.2 s following the stimulus onset. In Fig. 2b, each bar represents the change in the current in response to US, relative to the current in the immediately preceding recording in which no US was applied. There was no difference in the effects across the three applications (repetitions) of the US (*p* = 0.98, 1-way ANOVA). Thus, the effects of US on the transmembrane currents were robust and repeatable.

**Figure 3:**
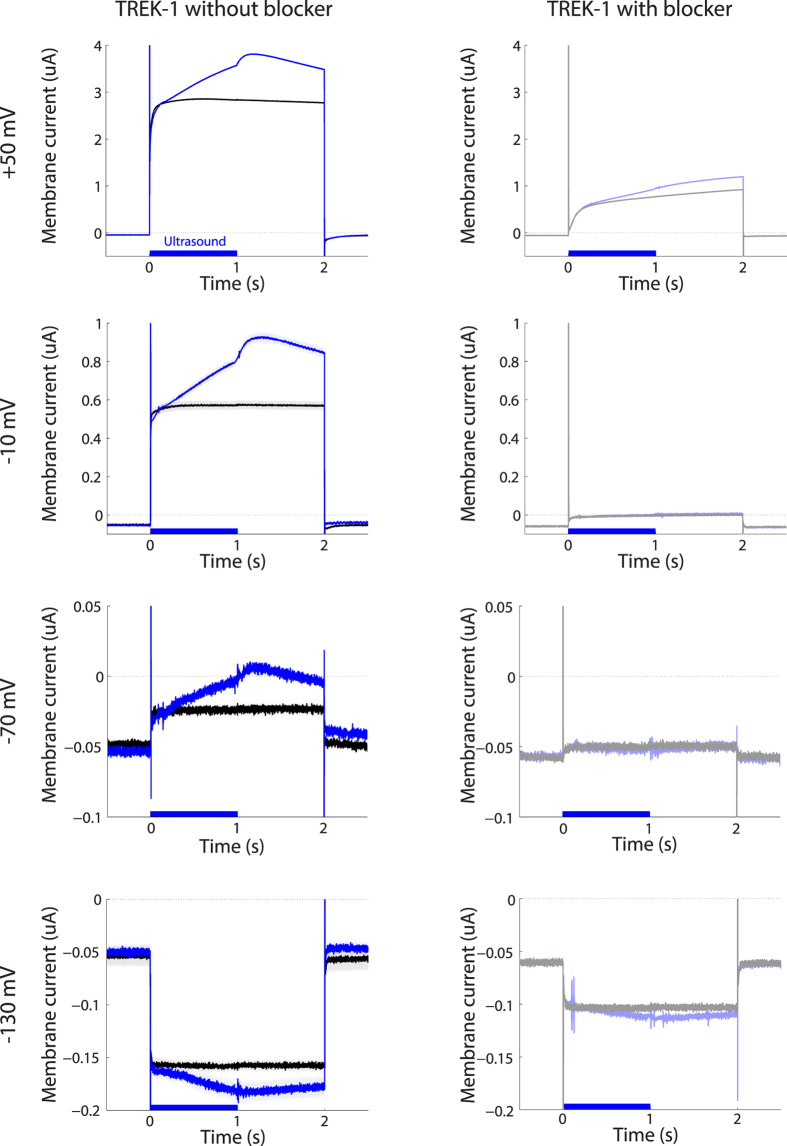
Ultrasound-mediated currents as a function of membrane voltage, in the absence and presence of TREK-1 blocker. Mean ± s.e.m. current flowing through the membrane of the example cell as a function of time. The mean was computed over the individual US (blue, *n* = 3) and noUS (black, *n* = 4) repetitions. The individual rows correspond to the currents measured at voltage steps of +50 mV, −10 mV, −70 mV, and −130 mV. In the data in the right column, same protocol sequence was repeated following the administration of BaCl_2_ (10 mM) into the bath.

Next, we tested the dependence of the US-induced currents on the membrane voltage. To test this, the initial membrane voltage of −80 mV systematically changed to one of four voltage levels: +50 mV, −10 mV, −70 mV, and −130 mV. Each level was maintained for 2 s before returning back to −80 mV. Again, at each voltage level, we alternated noUS and US iterations (4 and 3 repetitions, respectively). Figure 3 shows the currents flowing through the example cell in response to the US and the individual membrane voltages. At +50 mV (Fig. 3 top left), US increased the transmembrane current—measured in the same interval as previously—by 32.3% or 0.93 uA. Similar effects were observed at the other membrane voltages (Fig. 3, left column). In particular, at −10 mV, there was a 50.3% increase in the current. At −70 mV, the driving force on a potassium channel is small; yet, US had a strong relative effect on the transmembrane current even at this membrane voltage, increasing the current by 47.9%. The voltage −130 mV is below the potassium equilibrium potential. Consequently, for this voltage, one expects a negative (inward) current flowing through the K^+^ channels. This is indeed what we observed (bottom left). This voltage elicited an inward current (black). Furthermore, the effect of the US (blue) also reversed polarity; the US in this case increased the current in the inward direction. The US effect at this voltage amounted to 24.4% (note that the current percent *change* is positive also in this case, which will aid the interpretation of subsequent figures that show percent changes). Consistent with the notion that the currents were carried by TREK-1, the transmembrane currents were strongly suppressed following the administration of BaCl_2_ (10 mM), a K2P channel blocker^31^,^42^,^43^ into the bath (Fig. 3, right column).

**Figure 4:**
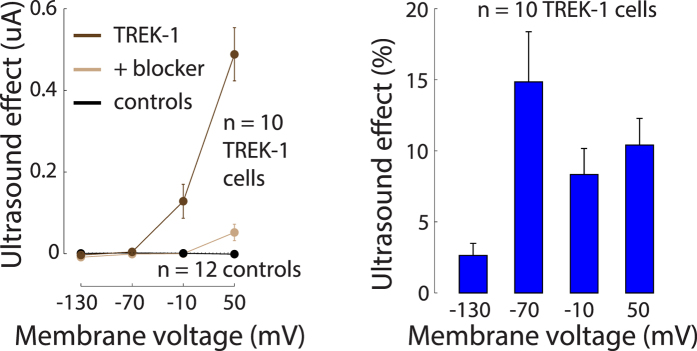
The effects of ultrasound on TREK-1 current. Left: Mean ± s.e.m. current due to US as a function of membrane voltage, for TREK-1 cells (dark brown; *n* = 10), the same TREK-1 cells with BaCl_2_ (10 mM) in the bath (light brown), and control cells (black; *n* = 12). The US stimulus pressure amplitude was 120 kPa, corresponding to *I*_SPTA_ ≈ 0.5 W/cm^2^. The currents were measured in the same interval as previously. Right: Mean ± s.e.m. relative changes in the transmembrane currents due to US, for the individual membrane voltages, quantified in the same interval as previously.

Figure 4 shows the currents due to the US for the individual membrane voltages and for all recorded cells (*n* = 10) at the pressure amplitude of 120 kPa (~0.5 W/cm^2^), measured in the same interval as previously. It has been found^44^ that mechanical effects on TREK-1 increase in an approximately exponential fashion with increasing membrane voltage. In accord with that study, we observed that also the effects of US on TREK-1 increase with increasing membrane voltage (Fig. 4, left). The US-mediated current changed polarity at *E*_*Kdata*_ = −104.2 mV (see Methods). This value aligned with K^+^ equilibrium, estimated to be *E*_*Kestim*_ ≈ −102.4 mV (Methods).

The addition of the BaCl_2_ K^+^ channel blocker into the bath greatly diminished the membrane currents due to the US (Fig. 4 left, light brown). Over the individual voltages, the blocker reduced the currents to at most 16.6% of the original current, and the ultrasound-mediated effects to at most 13.3% of the original effect. The reduction of the ultrasound-mediated effects was significant (two-way ANOVA with factors voltage and blocker on the current due to US: significance of blocker, *p* < 0.0001). Water-injected control cells (*n* = 12) showed very little modulation of transmembrane currents by the US (black). For these cells, at any membrane voltage, the effect did not exceed an average 2 nA modulation.

We quantified the US effects as the relative changes in the membrane current due to the US, i.e., as 100* (*I*_US_ − *I*_noUS_)*/I*_noUS_ (Fig. 4, right). The figure reveals that US modulated the membrane currents on average by 9.1%. An ANOVA detected a trend toward a modulation of the effect by membrane voltage (*p* = 0.044). However, the data at −70 mV should be considered with care because there are only very small currents flowing through the membrane at this voltage, and so estimates of relative current changes at this voltage are not necessarily accurate due to the division by small current.

**Figure 5:**
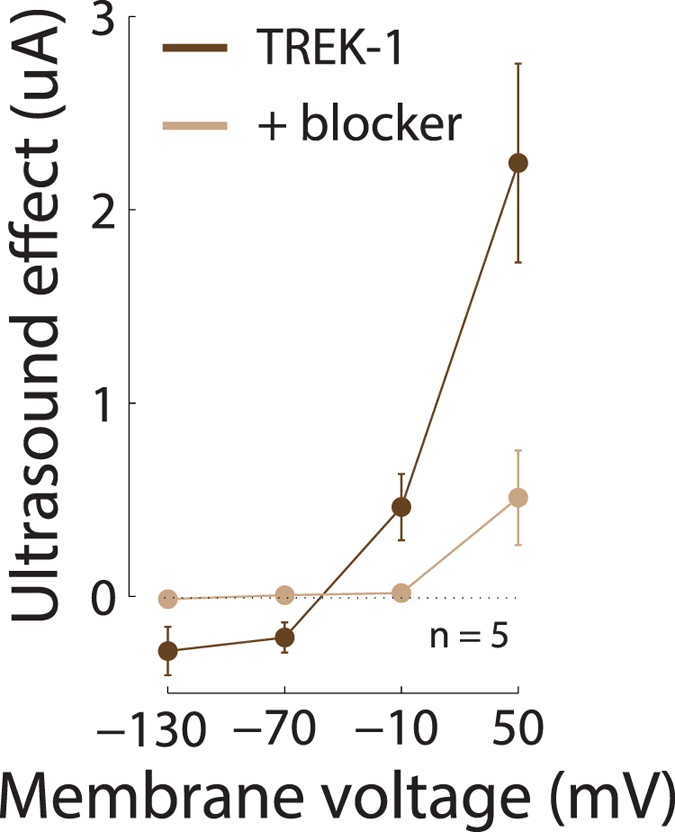
Ultrasound-mediated effects at elevated extracellular K^+^ concentration. Same format as in Fig. 4 left, for 5 TREK-1 cells recorded in ND96 with a 20 mM K^+^ concentration.

To further validate that the currents mediated by US were indeed K^+^ currents, we increased the concentration of extracellular K^+^ from 2 mM to 20 mM. This test shifts the K^+^ equilibrium from *E*_*Kestim*_ ≈ −102.4 mV to *E*_*Kestim*_ ≈ −44.8 mV (see Methods). Indeed, in a new set of TREK-1 cells recorded in this condition, we observed an according change in the reversal potential of the US effect (Fig. 5), which on average amounted to −51.1 mV. This result further corroborates the finding that US induced an increase in K^+^ currents.

The US-mediated currents exhibited the expected K^+^ reversal potential (Fig. 4 left, dark brown; Fig. 5), showed the characteristic voltage dependence of TREK-1 mechanosensitive currents (Fig. 4 left, dark brown), were greatly reduced following the application of the BaCl_2_ K^+^ channel blocker (Fig. 4 left, light brown), and no effects were observed in control cells (Fig. 4 left, black). These data suggest that US activated the TREK-1 ion channel.

**Figure 6:**
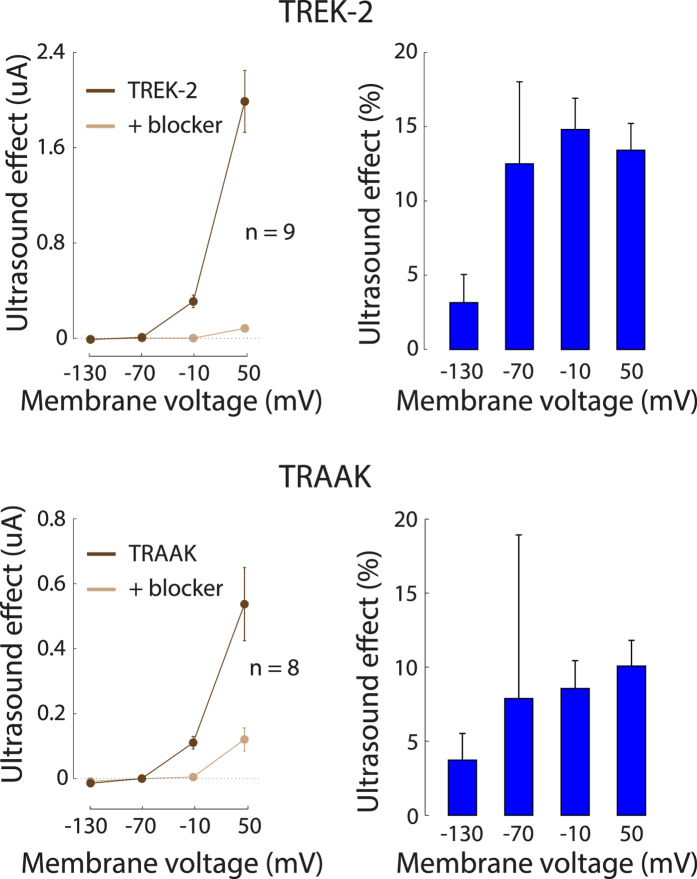
The effects of ultrasound on TREK-2 and TRAAK currents. Same protocol and format as for TREK-1 (Fig. 4). In the right panel (relative effects), the data can show a large s.e.m. for the −70 mV holding voltage due to the division by small currents at this voltage.

**Figure 7:**
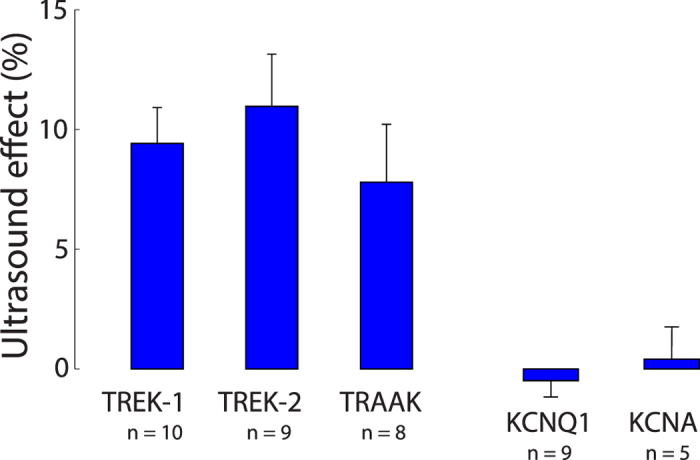
Summary of the effects of ultrasound on K2P channels. Mean ± s.e.m. effects averaged all holding voltages. The values are taken over the same interval as previously. The number of cells are shown below the channel labels.

We investigated the US responses of two other members of the K2P family of ion channels, TREK-2 (hK2P10.1, KCNK10) and TRAAK (hK2P4.1, KCNK4), using the same protocol. Cells expressing these channels showed very similar effects as cells expressing TREK-1 (Fig. 3). The TREK-2 (*n* = 9) and TRAAK (*n* = 8) cells exhibited a similar dependence of the US effect on membrane voltage as the TREK-1 cells (Fig. 6 left, dark brown), showed a comparable reversal potential (Fig. 6 left, dark brown), and were blocked by BaCl_2_ in a similar fashion as TREK-1 (Fig. 6 left, light brown). The relative effects (percent changes, Fig. 6 right) were weakly dependent on membrane voltage (ANOVA, *p* = 0.063 for TREK-2, *p* = 0.78 for TRAAK), and were comparable to that of TREK-1: the average effect over all voltages (Fig. 7) was an 11.0% modulation for TREK-2 and a 7.6% modulation for TRAAK. The effects were indistinguishable across the three channels (Fig. 7, ANOVA, *p* = 0.56).

We contrasted these effects with those of two other voltage-gated K^+^ channels, KCNQ1 and KCNA. Both reliably expressed in the oocyte (mean current, measured in the same interval as previously, for the +50 mV step: KCNQ1: 3.1 *μ*A; KCNA: 3.9 *μ*A). Yet, there was no significant modulation of the currents by the US (Fig. 7, *p* > 0.058 for any of the four holding voltages). This result suggests that US selectively activates ion channel families with specific properties.

**Figure 8:**
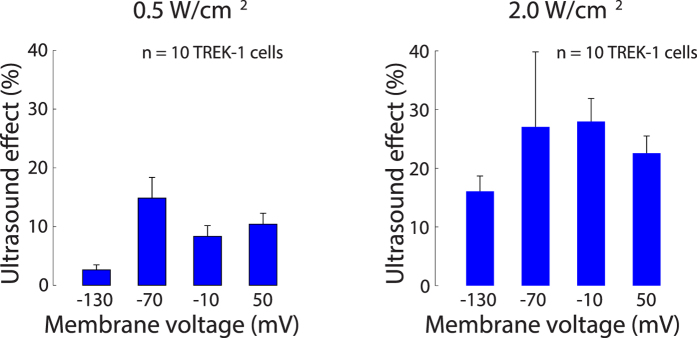
Ultrasound-mediated effects increase with stimulus intensity. Mean ± s.e.m. relative changes in the transmembrane currents due to US, for the individual membrane voltages, quantified in the same interval as previously. The data are shown separately for a lower (left) and a higher (right) stimulus intensity applied to separate sets of cells expressing TREK-1.

We next investigated how the effects of US depend on the US pressure. To do so, we recorded currents in a set of 10 TREK-1 cells at a pressure of 240 kPa (~2.0 W/cm^2^), and compared the relative effects to the weaker stimulus of 120 kPa (0.5 W/cm^2^) (Fig. 8). The figure demonstrates that the stronger stimulus evoked larger relative effects (average modulation of 23.4%) compared to the weaker stimulus (average modulation of 9.1%). The strongest average effect (27.9%) was observed at 240 kPa and at −10 mV; in this case, one cell exhibited a 50.3% and another cell a 44.1% current increase due to US. We assessed the significance of this difference and of the individual voltages using a two-way ANOVA. The ANOVA identified a significant effect of the pressure level (*p* < 0.001); the relative US effects over the individual voltages were not significantly different (*p* = 0.15). These data demonstrate that the US-induced K^+^ current is a function of the US stimulus strength.

**Figure 9:**
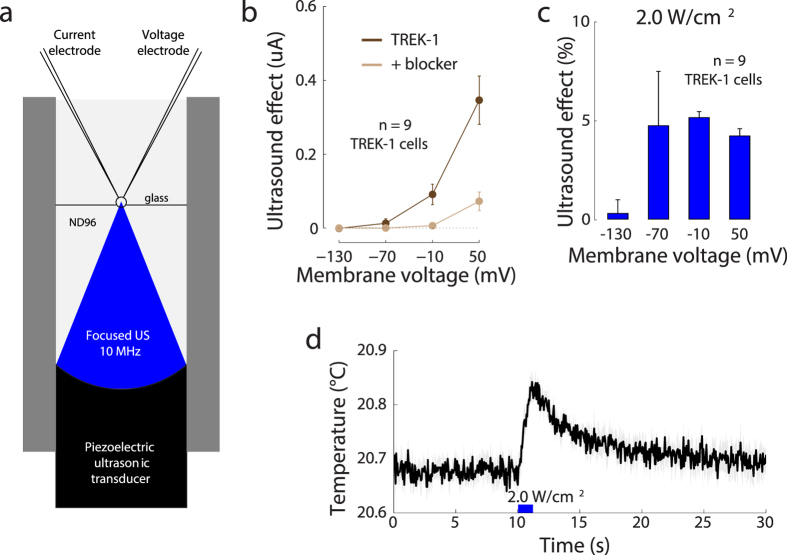
The effects of US on TREK-1 current when US propagates directly into the cell. (**a**) An alternative recording setup in which a cell is placed into a 0.7 mm opening within two thin sheets of borosilicate glass. Both the cell and the transducer share the same ND96 bath. (**b**) Mean ± s.e.m. current due to US (*n* = 9 cells) as a function of the membrane voltage, for the TREK-1 cells (brown) and TREK-1 cells with BaCl_2_ (10 mM) in the bath (light brown). Same format as in Fig. 4. (**c**) Mean ± s.e.m. relative changes in the transmembrane currents due to the US, for the individual membrane voltages. Same format as in Fig. 4. (**d**) Mean ± s.e.m. temperature (*n* = 5 recordings) of cell membrane surface measured by an infrared camera. The 10 MHz 240 kPa (2.0 W/cm^2^) US stimulus was applied at 10 s, and lasted for 1 s (thick blue bar).

Next, we investigated how the results reported thus far depend on a particular recording setup. The presence of a cell in a solid container may involve three issues. First, the bottom of the solid container, in response to US, might exert mechanical forces on the cell, which may not be present in biological tissues. Second, US might increase the temperature of the substrate, which may influence the activity of the channels. Third, the presence of a substrate may influence the amount of US radiation force experienced by the cell. To circumvent these issues, we devised an additional design in which the US propagates directly into the cell without having to pass through an intermediate solid object. In this setup (Fig. 9a), a cell is positioned within a 0.7 mm opening created by two thin sheets of borosilicate glass. The cell and all inner components of the setup are immersed in the ND96 recording solution. In this design, the narrow beam of the 10 MHz focused US propagates through the recording solution directly into the cell. In this setup, US (240 kPa, 2.0 W/cm^2^) again induced current changes over all tested voltages. The effects were weaker but otherwise reproduced those observed with the former setup (Fig. 9b,c). The US-induced currents (Fig. 9b, dark brown) crossed zero at −116.5 mV, a value similar to that observed in Fig. 4. Also as shown previously, the K^+^ channel blocker (Fig. 9b, light brown) greatly reduced the US-mediated currents. Thus, US elicited K^+^ current flowing through the TREK-1 ion channel also in this modified setup, and so a presence of a solid substrate below a cell is not required to observe an US effect on ion channel currents.

The 240 kPa (≈2.0 W/cm^2^) stimulus might be strong enough to increase membrane temperature. We measured the membrane temperature in this setup using a high-sensitivity infrared camera (see Methods). The testing conditions were identical to those during the data collection, with the exception that the test cell was only partially immersed in the ND96 solution. This allowed us to measure the cell’s surface temperature using the infrared camera positioned above the cell. Just like during the data collection, we applied the 240 kPa 10 MHz stimulus for a period of 1 s. Figure 9d shows the average temperature of the membrane surface as a function of time. The US is applied during the time indicated by the blue bar. At the end of the 1 s application of the US, there was a Δ*T* = 0.15 °C increase in the temperature compared to the 10 s period preceding the US onset. This increase was significant (t-test, *t*_4_ = 9.43, *p* < 0.001, *n* = 5 measurements). This value is within the range of that estimated theoretically for this US pressure and duration assuming water constitution (see Methods)—the estimate suggests a change of 0.10 °C. It is likely that the absorption coefficient of the cell is higher compared to that of the assumed water, and so the actual increase in temperature can be somewhat higher than the theoretical estimate. There was no significant temperature increase (Δ*T* = −0.01 °C, *p* = 0.46) when the cell was replaced by a thin sheet of borosilicate glass with its top part unexposed to water. Although a change of Δ*T* = 0.15 °C may seem negligible from the perspective of biological safety, this effect may, at least in part, contribute to the mechanism by which US mediates the TREK-1 opening. Around the room temperature of 20 °C, an increase from 15 °C to 25 °C (a change of 10 °C) about doubles (≈100% increase) the current flowing through TREK-1^45^. Consequently, a change of Δ*T* = 0.15 °C could lead to a 1.5% increase in the TREK-1 current. This value is smaller than the ≈5% effect observed in the data in this case, but could account for a portion of the effect.

Recent studies of the effects of US on neurons often report cellular excitation^2^,^4^,^6^.The excitation may be mediated by voltage-gated sodium channels^2^,^4^. We investigated the response of Na_V_1.5 (SCN5A), a Na^+^-selective voltage-activated ion channel expressed in cardiomyocytes, neurons, and other excitable cells^34^,^35^,^36^,^37^. This ion channel has been shown to be sensitive to mechanical stimuli^36^,^38^,^39^,^40^ and might therefore respond also to US. We expressed this channel with the β1 subunit, which has been shown to increase peak currents and accelerate recovery from inactivation^46^,^47^. This allowed us to systematically test the channel’s response to a range of holding voltages and a range of stimulus intensities (see Methods). To implement the protocol of a study showing sensitive US responses^2^, we pulsed the US at a frequency of 1 kHz with 5% duty (see Methods). We used the setup shown in Fig. 9a.

**Figure 10:**
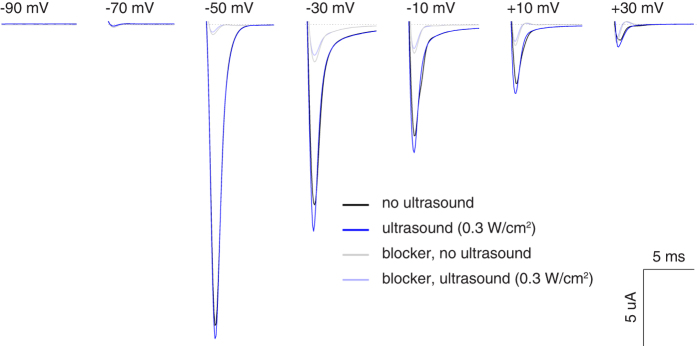
The effects of US on Na_V_1.5 current. Average membrane current of a cell expressing Na_V_1.5 *α* and *8*_1_ subunits in response to 20 ms voltage steps of a particular value (see inset). We alternated 4 noUS and 3 US conditions; average noUS (black) or US (blue) current is shown. Pulsed ultrasound (10 MHz, 1 kHz pulse repetition frequency, 50 us pulse duration, *I*_SPTA_ = 0.3 W/cm^2^) was applied throughout the US condition (see Methods). The light traces show corresponding currents following the introduction of ranolazine (500 *μ*M), a Na_V_1.5 channel blocker, into the bath.

Figure 10 shows the transmembrane currents of an example cell expressing Na_V_1.5. The cell was subjected to 20 ms depolarizing steps to voltages indicated in the figure. In accord with previous findings, when no US was applied (black), the cell showed robust currents that emerged for steps to voltages greater than −70 mV and gradually decreased in amplitude with increasing voltage^38^. Crucially, the application of US (*I*_SPTA_ = 0.3 W/cm^2^) led to an increase in the average current flowing into the cell during all voltage steps greater than −70 mV (blue). This effect was robust and repeatable: as in the protocol for K2P channels, we strictly alternated noUS and US conditions and thus accounted for any possible gradual effects. The application of 500 *μ*M ranolazine, an Na_V_1.5 channel blocker^40^, greatly reduced the currents (light colors) and also abolished the US effects. This is in line with a previous report of ranolazine mitigating effects of mechanical stimuli on Na_V_1.5^40^.

**Figure 11:**
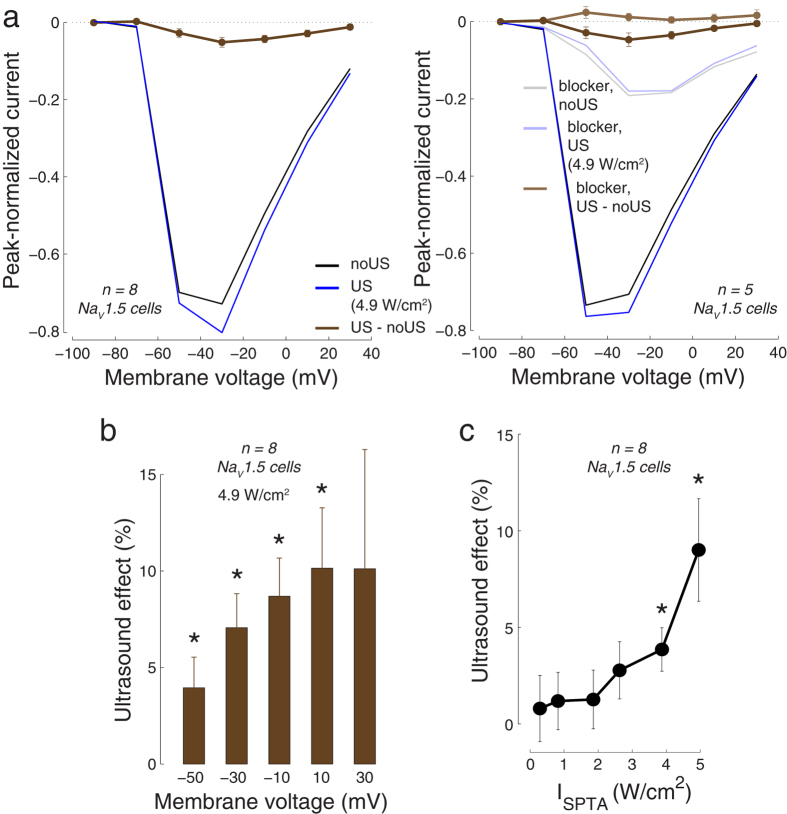
Summary of the effects of US on NaV1.5 currents. (**a**) Right panel: Average peak negative current in Na_V_1.5-expressing cells (*n* = 8) in response to voltage steps (same as in Fig. 10), separately for the conditions in which there was no US (black) and in which US was applied (blue). The currents of each cell were normalized by the cell’s overall peak negative current before computing the average over cells. For each cell, we alternated 4 noUS and 3 US conditions, and took the average within each condition. The brown curve shows the mean ± s.e.m. difference between the US (blue) and noUS (black) currents. Left panel: Same as right panel for the cells that in addition had ranolazine blocker data (*n* = 5). The currents following the application of ranolazine (500 *μ*M) are shown in light colors. (**b**) Mean ± s.e.m. relative effects of US (100 * (*I*_US_*-I*_noUS_)*/I*_noUS_) for the individual voltages. The figure shows effects for voltages in which currents were substantial to circumvent division by a small number (small currents at −90 mV and −70 mV). Both (**a,b**) used stimulus intensity *I*_SPTA_ = 4.9 W/cm^2^. *p < 0.05, two-sided t-test, *n* = 8. (**c**) The effects of US as a function of stimulus intensity. The effects were averaged over the voltage steps; mean ± s.e.m. values over cells (*n* = 8) are shown. *p < 0.05, two-sided t-test.

We quantified these effects for all recorded cells by measuring the peak negative current for each voltage step (Fig. 11). In this figure, the currents of each cell were normalized (i.e., divided) by the cell’s peak negative current. As with the example cell, the cells showed expected responses to the membrane voltages steps (Fig. 11a left, black), and there was an increase in the inward current in response to US (4.9 W/cm^2^; blue). The effect of ultrasound (brown), i.e., the difference between the US (blue) and noUS (black) currents, was significant for the −50, −30, −10, and 10 mV voltage steps (*p* < 0.05, paired two-sided t-test, *n* = 8). Similar effects were observed in a proportion of cells (*n* = 5), which in addition had ranolazine data (Fig. 11a, right): these cells showed significant effects for the −30, −10, and 10 mV steps (dark brown). As with the example cell, ranolazine greatly reduced the inward currents (light black, light blue), and abolished the US effects (light brown, *p* > 0.08 for all voltages).

We quantified the US effects as percent changes (100* (*I*_US_*−I*_noUS_)/*I*_noUS_) for all voltages in which the cells showed substantial current (Fig. 11b). The effects were modest (a 4–10% current increase), but significant for the −50, −30, −10, and 10 mV voltage steps (*p* < 0.05, two-sided t-test, *n* = 8). In line with studies investigating the effects of the strength of mechanical stimuli on Na_V_1.5^36^,^39^,^40^, we also found that the effects of US increased with increasing stimulus strength (Fig. 11c). For the stimulus of 4.9 W/cm^2^, there was an average 8.7% current modulation (*p* = 0.019, two-sided t-test, *n* = 8).

Together, these data suggest that US induced small but significant responses of the Na_V_1.5 ion channel.

## Discussion

US has emerged as a new non-invasive way to stimulate neuronal or retinal circuits. In comparison to other non-invasive modalities such as the TMS or tDCS, US can be relatively well focused, and the focal point can be located deep in the brain due to the favorable propagation properties. This approach provides the opportunity to non-invasively and flexibly stimulate specific regions of the brain, which lends itself to important applications. However, such applications are impeded by our current lack of understanding of the mechanism by which US conveys the neuromodulatory effects. It has been hypothesized that US may influence the activity of mechanosensitive ion channels embedded within cellular membranes. In a controlled experiment, we found that focused US elicits transmembrane currents flowing through mechanosensitive K^+^ and Na^+^ ion channels at intensities used previously to activate retinal cells or neurons^6^,^8^.

The finding that US modulates K^+^ currents of K2P channels, which are expressed in the brain and in the retina^27^,^28^,^29^,^30^,^31^, may provide a possible mechanism for the findings that US can inhibit neuronal activity^3^,^7^,^9^,^48^. In addition to the inhibitory effects, these channels might also indirectly mediate an excitation. For instance, US may activate K2P channels in retinal cells and so lead to an outward, hyperpolarizing (inhibitory) K^+^ current. Because US was found to act on inhibitory cells within the retina^6^, the inhibition of these inhibitory cells may lead to the observed cellular excitation^6^. As another example, TREK-1 is particularly abundant in GABA-containing interneurons of the caudate nucleus and putamen^30^. An activation of these neurons using US may result in an excitation of the cells these interneurons project to.

Cellular excitation by US might be directly mediated by an activation of Na^+^ channels^2^,^4^. Our finding of a modulation of Na_V_1.5 currents by US supports this hypothesis. Nonetheless, the effect was modest (~9% modulation) given a strong stimulus (~5 W/cm^2^), and it is therefore unclear whether this mechanism could lead to a generation of action potentials. Furthermore, these effects should be interpreted with care because Na^+^ currents recorded by the two-electrode voltage clamp are at least in part influenced by capacitive transients and an incomplete voltage clamp. There are, nonetheless, three indications of a specific US effect on the Na_V_1.5 currents. First, the effect of US was maximal in the voltage range between −30 mV and −10 mV (Fig. 11a, brown), consistent with previous reports of voltage sensitivity of this channel. Second, the effect was mitigated with ranolazine, a Na_V_1.5 channel blocker (Fig. 11a right, light brown). Third and finally, the effect of US was a function of the stimulus intensity, attaining significance specifically for higher stimulus intensities (Fig. 11d). Together, the effects of US on Na_V_1.5 reported here may serve as a starting point for future targeted investigations of the effects of US on currents flowing through Na^+^ channels.

Our findings provide a possible resolution to the contradictory findings that US may elicit inhibitory^3^,^7^,^9^,^48^ or excitatory^2^,^4^,^6^ effects. In particular, if cells in a given tissue express predominantly Na^+^ or Ca^++^ ion channels responsive to US, US may lead to cellular excitation. Reversely, US may lead to an inhibition in cells that express predominantly K^+^ US-sensitive ion channels. According to this framework, US effects may interact or even cancel out in cells that show a fine balance of excitatory and inhibitory ion channels. In this regard, future studies should carefully investigate the timing of the US effects for each US-responsive ion channel.

The reported effects of US on ion channels may not be the only way how US activates neurons or retinal cells. Heating, cavitation^8^, or mechanical effects that do not act on ion channels—such as changes in membrane capacitance^22^,^23^, may play a role in the reported effects on neurons. Isolating the contribution of each such factor should be a central theme of future inquiry.

We only scratched the surface of the possible US parameters and protocol properties. US intensity, carrier frequency, pulse repetition frequency, duty cycle, and the applied geometry likely all factor in eliciting an optimal response. On this front, we attempted to work also with a 500 kHz stimulus. At this frequency, there were significant effects on TREK-1 already at a relatively low US pressure of 40 kPa in amplitude. However, the 500 kHz stimulus induced noticeable leak currents in the recordings, substantially shifting the reversal potential in the positive direction (about half way toward zero). This could have been due to a resonance of the recording electrodes at this low US frequency^4^. Because such leak occurred in a majority of the recorded cases, we do not report these data in this study. With regard to Na_V_1.5, US duration and the period necessary to recover from inactivation may also influence the magnitude of the US effects. Future studies should subject each channel candidate to a combination of US and recording parameters and determine which parameter set leads to an optimal response. In particular, in this study, we worked with relatively mild stimuli to mitigate the possibility of heating (Fig. 9d). Future studies should investigate the effects of also stronger stimuli, as our data indicate that the ion current effects increase with increasing stimulus intensities (Figs 8 and 11c).

This study establishes a first step toward characterizing the effects of US on ion channels at the single cell level, demonstrating that US application without cavitation can modulate currents flowing through specific ion channels. Our approach eliminates many confounding factors that would be present in a more complex biological system such as an *in vivo* brain. However, an outstanding question is how well this setup models effects in neuronal tissues. Cells in neuronal tissues have elastic ties with their neighbors, are substantially smaller, and likely have different cytoskeletal composition than that of the *Xenopus* oocyte. Follow-up experiments must therefore compare the effects reported here with those obtained in tissue cultures or slices.

Many ion channels in the brain and the heart might respond to mechanical- or temperature- related effects associated with the application of US^20^,^26^,^43^. Our findings that US activates channels of the K2P family and a voltage-gated sodium ion channel suggest that US may be capable of activating other ion channels as well. In particular, US may activate other members of the K2P family^49^, other voltage-gated Na^+^ channels^36^, voltage-gated Ca^++^ channels^4^, acid- sensing ion channels (ASICs)^50^, TRP channels^51^, or channels of the recently characterized Piezo family^52^,^53^,^54^, among others^50^.

The finding that US can influence ion channel activity may lead to the development of new non-invasive ways to control the activity of specific cells. Such tool may greatly benefit basic research. In such an approach, an existing or an engineered US-sensitive ion channel could be over-expressed in a particular region or in cells carrying specific genetic markers. US would then be used to non-invasively excite or inhibit these cells, depending on the channel type, at an intensity that is lower than that necessary to activate native ion channels. Such “sonogenetic” approach would be analogous to optogenetics^55^,^56^, but would confer the advantage of the noninvasive propagation of US through the skull. Thus, no surgery would be required to control cellular discharge in transgenic animals expressing the specific US-sensitive ion channel in particular regions or cell types. An analogous “thermogenetic”^57^,^58^,^59^ approach might use US to activate ion channels sensitive to temperature.

In summary, we devised a direct way to study the effects of US on ion channels. We found that focused US modulates K^+^ currents of K2P channels and Na^+^ currents of Na_V_1.5. These ion channels are expressed in neurons, retinal cells, heart, and other tissues. An effect of US on these ion channels may therefore constitute one of the mechanisms underlying the reported effects of US on retinal cells and neurons. The finding opens doors to future investigations of the effects of specific US parameters on ion channels. A demonstration of a safe, reversible mechanism of US action on cellular excitability might have important implications to several medical fields. This finding also paves the way to work on sonogenetics and thermogenetics—ultrasound-based, non-invasive analogues of optogenetics.

## Methods

### Channel expression

We synthesized the channel cRNA using the mMessage T7 polymerase kit (Applied Biosystems). The cRNA (TREK-1 (hK2P2.1, KCNK2): 2 ng; TREK-2 (hK2P10.1, KCNK10): 0.5 ng; TRAAK (hK2P4.1, KCNK4): 40 ng; Na_V_1.5 (SCN5A): 40 ng, equal ratio of *α*:*β*_1_ subunits; KCNQ1 (KvLQT1): 8 ng; KCNA (*Drosophila* Shaker): 8 ng) was microinjected (Drummond Nanoject, Broomall, PA) into stage V or VI defolliculated *Xenopus* oocytes. Injected cells were incubated in ND96 solution (96 mM NaCl, 2 mM KCl, 1.8 mM CaCl2, 1 mM MgCl2, 5 mM HEPES, pH 7.6) at 18 °C for 2–5 days before performing the experiments.

### Setup and ultrasound application

A tone burst ultrasound wave was generated using an immersion focus ultrasonic transducer, V327-SU (Olympus), which had a focus at 25.4 mm (1 inch), nominal element size of 10 mm, and center frequency of 10 MHz. A single oocyte was positioned either on a plastic plate (8 mm in diameter, 5 mm in depth, 0.5 mm bottom thickness), or within an opening between two thin sheets of borosilicate glass (100 um thickness; Warner Instruments); in both cases in the center of the US focus. The bath was filled with ND96. The recording chamber was placed atop of a water container that housed the US transducer. The transducer was submerged in deionized and degassed water, which provided acoustic coupling to the cell. The walls were made of modeling clay. The clay was chosen as a material that provided tight seal and that serves to absorb possible ultrasonic vibrations or reflections. The output pressures were measured using a calibrated hydrophone (HGL-0200, Onda, Sunnyvale, CA) combined with a pre-amplifier (AG-2020, Onda, Sunnyvale, CA). The hydrophone measurements were performed at the peak spatial pressure. When converting the measured voltages into pressures, we accounted for the hydrophone and pre-amplifier capacitances according to the manufacturer’s manual (Onda, Sunnyvale, CA). In a proportion of the recorded cells (6 out of the 10 cells at 240 kPa), the ND96 recoding bath was capped with a neoprene absorber (5 mm in thickness and 10 mm in height) to prevent the formation of a standing wave. The results with and without the absorber were similar and so we did not distinguish between the two cases.

The transducers were driven using a signal generator (33220A, Agilent) connected to a broadband amplifier (75A250, Amplifier Research). The timing of the US stimuli was controlled by the HEKA Pulse software (HEKA, Germany) interfaced with an ITC-16 ADC board.

### Electrophysiological recordings

The transmembrane currents were measured using the two-electrode voltage clamp^60^. Glass microelectrodes were pulled with impedance between 0.5 and 5 MΩ and filled with 3 M KCl solution. The two recording electrodes and the recorded cell were immersed in the ND96 solution. The voltage clamp was controlled by the Geneclamp 500B device (Axon Instruments). The device low-pass filtered the measured currents at 2 kHz to prevent aliasing. Signals were acquired at 10 kHz by the ITC-16 board and maintained within the HEKA Pulse software (HEKA, Germany). The recorded traces were notch filtered using a 60 Hz IIR 0-lag filter (applied using the filtfilt function in Matlab).

### Experimental protocols

In the experiments with K2P channels, cells were held at a resting voltage of −80 or −70 mV. After 0.5 s, the voltage stepped to +50, −10, −70, or −130 mV. This membrane voltage was maintained for 2 s, before returning back to the resting voltage. Within each block of one of the four membrane voltages, we alternated conditions in which US was applied and in which it was not. The individual conditions of the sequence (#1: noUS, #2: US, #3: noUS, #4: US, #5: noUS, #6: US, #7: see Fig. 2) followed in quick succession, with a 5 s period in between. The US onset coincided with the voltage step. The US was applied as a continuous (10 MHz) sine wave for a period of 1 s. After the sequence of the 7 repetitions was completed for a given voltage, the voltage changed to one of the other voltages. Thus, a total of 7 * 4 = 28 iterations were recorded. Following this block, 10 mM BaCl_2_ was administered into the bath. BaCl_2_ was chosen for its relatively strong blocking effects^31^,^42^,^43^. The same sequence of 28 iterations was performed with BaCl_2_ in the bath. Thus, each experiment comprised a total of 56 iterations.

In the experiments with the Na_V_1.5 channel, cells were held at a resting voltage of −90 mV. After 40 ms, the voltage stepped to −70 mV for 20 ms, and returned to −90 mV for 20 ms. This was repeated in sequence for steps to −50, −30, −10, 10, and 30 mV, in each case returning to −90 mV. This sequence of voltage steps was repeated 5 times, spanning a total time of 1400 ms. The effects were similar within each of the 5 sequences, and so we averaged the measured currents over the 5 sequences. The US stimulus (same transducer, 10 MHz, pulsed at 1 kHz at 5% duty (50 us pulse width), time-average stimulus intensities ranging from 0.3 to 4.9 W/cm^2^ (see Stimulus pressure and intensity)) was turned on 20 ms before the first step and turned off 20 ms following the last step. As in the protocol with K2P channels, we strictly alternated noUS and US conditions (4 noUS and 3 US) and averaged currents within the corresponding condition. The Na_V_1.5 experiments were performed exclusively with the setup featuring an opening between two thin sheets of borosilicate glass (Fig. 9a).

### Stimulus pressure and intensity

A given US pressure measured at the cell can be converted into stimulus intensity (average power per unit area) according to:


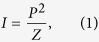


where *P* is the effective pressure (which for a sine wave is about 0.707 of the pressure amplitude) and

*Z* is the acoustic impedance of a cellular membrane (*Z* ≈ 1.55* 10^6^ kg/m^2^/s in soft biological tissues^61^).

Using this conversion, for a continuous stimulus, pressure amplitudes of 120 kPa and 240 kPa correspond to about 0.5 W/cm^2^ and 2.0 W/cm^2^. In the experiments with Na_V_1.5 (Fig. 11), we used pressure amplitudes ranging from 425 kPa to 1.75 MPa. In this case, the US was pulsed at 1 kHz with 5% duty (i.e., 50 us pulse width), which results in time-average intensities ranging from about 0.3 W/cm^2^ to about 4.9 W/cm^2^.

### Reversal potential

The K^+^ reversal potential *E*_*K*_ was measured using linear interpolation of the US-mediated currents between −130 mV and −70 mV. *E*_*K*_ was taken as the voltage for which the interpolation line crossed zero. In the ND96 solution and at room temperature, assuming an intracellular K^+^ concentration of 120 mM, *E*_*K*_ is estimated to be *E*_*Kestim*_ ≈ 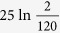 ≈ −102.4 mV. In the experiment with an extracellular K^+^ concentration of 20 mM (Fig. 5), *E*_*K*_ is estimated to be *E*_*Kestim*_ ≈ 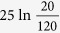 ≈ −44.8 mV.

### Estimation and measurement of temperature

The application of an ultrasonic stimulus of target effective pressure *P* over time Δ*t* changes target temperature Δ*T* according to^8^,^62^:


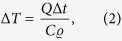


where *Q* is the heat generated by US beam in the medium, Δ*t* is the US duration (Δ*t* = 1 s), *C* is the specific heat capacity of the medium (*C* ≈ 3600 J/kg/K), and *ϱ* is the density of the medium (*ϱ* ≈ 1028 kg/m^3^). The heat *Q* generated by the US field can be calculated^62^ as


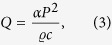


where, in addition to the above, *c* is the speed of US in the medium (*c* ≈ 1515 m/s), and *α* is the absorption coefficient (*α* ≈ 20 m^−1^ for the frequency of 10 MHz^63^). The pressure *P* is the effective value of the US field (~0.707 of the pressure amplitude).

We also measured the temperature of the cell membrane using a sensitive infrared camera (SC5000, FLIR systems). To prevent an absorption of the infrared signal by water, only the bottom part of the measured cell was immersed in the ND96 fluid; the top part was in direct contact with air. The cell was positioned 5 cm below the head of the camera, in accord with the camera use guidelines.

## Additional Information

**How to cite this article**: Kubanek, J. *et al*. Ultrasound modulates ion channel currents. *Sci. Rep.* **6**, 24170; doi: 10.1038/srep24170 (2016).
